# Complex adaptive learning cortical neural network systems for solving time-fractional difference equations with bursting and mixed-mode oscillation behaviours

**DOI:** 10.1038/s41598-023-48873-0

**Published:** 2023-12-17

**Authors:** Yu-Ming Chu, Saima Rashid, Taher Alzahrani, Hisham Alhulayyil, Hatoon Alsagri, Shafiq ur Rehman

**Affiliations:** 1 Department of Mathematics, Huzhou University, Huzhou, 313000 China; 2 Department of Mathematics, Government College University, Faisalabad, 38000 Pakistan; 3 Department of Computer Science and Mathematics, Lebanese American University, Beirut, 11022801 Lebanon; 4 College of Computer and Information Sciences, Imam Mohammad Ibn Saud Islamic University (IMSIU), Riyadh, 11432 Saudi Arabia

**Keywords:** Neurology, Engineering, Mathematics and computing

## Abstract

Complex networks have been programmed to mimic the input and output functions in multiple biophysical algorithms of cortical neurons at spiking resolution. Prior research has demonstrated that the ineffectual features of membranes can be taken into account by discrete fractional commensurate, non-commensurate and variable-order patterns, which may generate multiple kinds of memory-dependent behaviour. Firing structures involving regular resonator chattering, fast, chaotic spiking and chaotic bursts play important roles in cortical nerve cell insights and execution. Yet, it is unclear how extensively the behaviour of discrete fractional-order excited mechanisms can modify firing cell attributes. It is illustrated that the discrete fractional behaviour of the Izhikevich neuron framework can generate an assortment of resonances for cortical activity via the aforesaid scheme. We analyze the bifurcation using fragmenting periodic solutions to demonstrate the evolution of periods in the framework’s behaviour. We investigate various bursting trends both conceptually and computationally with the fractional difference equation. Additionally, the consequences of an excitable and inhibited Izhikevich neuron network (INN) utilizing a regulated factor set exhibit distinctive dynamic actions depending on fractional exponents regulating over extended exchanges. Ultimately, dynamic controllers for stabilizing and synchronizing the suggested framework are shown. This special spiking activity and other properties of the fractional-order model are caused by the memory trace that emerges from the fractional-order dynamics and integrates all the past activities of the neuron. Our results suggest that the complex dynamics of spiking and bursting can be the result of the long-term dependence and interaction of intracellular and extracellular ionic currents.

## Introduction

Firing neuronal cell frameworks are arithmetic quantifications of nerve cell properties implemented for describing physiological behaviours. Numerous initiatives have been rendered for modelling neuronal activity prospectively^1,2^. Actually, the overarching objective of the supplied neuron framework is to resemble neurological collaborative behaviours in an interactive setting. Plenty of research in neural networks (NNs) is underway to investigate their intricate behaviours involving synchronization^3,4^. The capability of the representation to display the typical behaviour of neuronal activity and its productiveness are actually the criteria employed for distinguishing among various NNs^5^. Hodgkin and Huxley’s (HH) chaotic formulae were initially used to demonstrate how the neurons electrical activity is associated with the propagation of voltages within the cellular membrane of the squid’s enormous arteries^6^. Multiple simplified versions of the HH framework that include the FitzHugh-Nagumo approach have subsequently been implemented. The absorbed and shoot systems constitute two of the increasingly frequently utilized frameworks for clarifying the characteristics of enormous neuronal systems^7^. These mathematical simulations are important for comprehending the functioning of a nervous framework; nonetheless, the biological process is excluded^5^. The Izhikevich neuron network (INN) system is a two-variable system that is extensively used in NN modelling. This framework attempts to replicate the majority of identified spikes in neurons in the cortex. The positive aspect of the INN system is that it examines physiological ideas, analogous to the HH approach, and is technically efficient, identical to the unified and bursting models^8^.

Recently, the essential theoretical and accurate estimation provided by fractional-order (FO) patterns^9–11^ of activated mechanisms to scientific neuron algorithms may serve an essential part in the effective acquisition and transmission of neurological facts. It can generate a variety of memory-dependent neurological procedures over numerous time frames^12,13^. The neurons located in the NN generate multiple spiking-bursting procedures for analyzing data and exchanging signals. For studying the temporal fluctuations of cell power, the generalization of integer-order systems is better suited and more productive. Brain neurons are responsible for memory. Because of the memory impact, FO dynamical structures can be implemented in this particular instance. Memory influence may additionally be utilized in an additional manner by permitting the implications of electromagnetic radiation and fields^14–16^. However, the INN model is a simple model that generates several types of neuronal responses. It is bio-physically plausible and computationally efficient. The system consists of two variables with FO dynamics. Although we mostly used the same FO for both variables, one can use different FOs that can vary on the interval (0, 1]. For fractional order close to 1, there was an attempt to implement FO-INN model^17^, but the article was a short report without significant results nor details.

Xi et al.^18^ suggested the finite-time robust control of uncertain FO Hopfield NNs via sliding mode control. The relevant articles mentioned above indicate the substantial popularity of using a Caputo-type fractional difference operator^19^ in the modeling of complex systems via time discreteness. Because DFC has numerous benefits over classical calculus, we need to employ it for investigating NN architectures^20,21^. For a pair of explanations, the modeling of NNs with fractional exponents can be utilized for studying neurons in biology. Initially, by boosting a level of liberation, the FO improves mechanism efficiency^20,21^. The memory and heridatory characteristics of several scientific models can be described using the variable-order (VO) fractional operators and their non-stationary power-law kernel. As a consequence, VO discrete fractional calculus (VODF) was available as an intriguing option for supplying an efficient computational scheme for precisely characterizing multifaceted natural mechanisms and procedures^22^. Following that, VO-FDEs have captured growing interest owing to their appropriateness in modeling a wide range of occurrences, such as anomalous diffusion^23,24^, viscosity mechanisms^25,26^, automation^27^, chemical engineering^28^, and numerous additional areas of science and technology^29,30^.

In 1993, Samko and Ross^31^ suggested the idea of VO integral and differential, in addition to certain fundamental features. Lorenzo and Hartley^32^ summarized the VO fractional operator study outcomes and subsequently examined the terminology of VO fractional operators in multiple configurations. Following that, some innovative, essential and significant implementations of the capabilities of the VO-FDE frameworks were additionally investigated in Refs.^33–39^.

Following the aforesaid propensity, we aim to investigate and analyse the neurological firing and exploding processes of the DFO-INN model for an extensive variety of DFOs, such as commensurate, non-commensurate and variable-order DFOs. It also provides a classical framework that assists with productive data analysis, stimulation apprehension and frequency-independent transitions of oscillating neural activity. Furthermore, the evolution in blasting position and spiking rate is examined using DFO modifications. It generates broad spikes and explosions by differing merely in the DFOs, while the rest of the factors stay steady. These different types of oscillation patterns can be produced by varying only the DFOs while all other parameters are fixed. Thus, the spiking and bursting dynamics that may be generated by systematic variations of several parameters can be controlled by the single parameter, i.e., the $\beta$. Decreasing the FO transforms the response of the random network of DFO-INN model from random to scale free pattern where a few neurons control the whole network. Consequently, FOs can control the various neuronal activation forms produced in a continuous-time INN system by deliberately altering multiple settings. Both stabilization and bifurcating assessments have been described for analyzing the structure and interactions of the DFO-INN model. Furthermore, reactions of an ensemble of DFO-INN (excitability and inhibitory) are studied to demonstrate the integrated interactions of the NNs at different FOs. The stabilization of the DFO-INN model is additionally fascinating to us. Another significant component of the INN model is synchronization, which is a technique that uses adaptive controlling variables to compel a slave system to maintain a similar track as a master. Additionally, we suggested an amalgamated synchronization tactic for the suggested DFO-INN model, in which the master is concurrently synchronized to a single slave framework. Our results suggest that the complex dynamics of spiking and bursting patterns controlled by several parameters can be the result of the long-term dependence and interaction of intracellular and extracellular ionic currents.

The remaining content of the article is structured as follows: The preliminary reports on the DFC and related postulates are presented in Section "Preliminaries on DFC". In Section "INN model and its FO formulation", we investigate the framework’s configuration in detail along with its biophysical mechanism. Section "Qualitative analysis of DFO-INN system" consists of fundamental dynamic features using quantitative and qualitative evaluations. Furthermore, we suggested adapted controllers in Section "Controlling dynamics of DFO-INN system" for stabilizing and synchronizing the chaotic pathways of the identified DFO-INN system. Ultimately, Section "Conclusion" presents the general paper’s final analysis.

## Preliminaries on DFC

Before we begin discussing chaotic DFC applications with stability and synchronization, we need to initially review certain of the relevant concepts. Within this section, it is necessary to refer^13^ to $\,^{c}\Delta _{\varpi }^{\beta }\digamma (\xi )$ the $\beta$-Caputo type delta difference of a mapping $\digamma (\xi ):\mathbb {N}_{\varpi }\mapsto \mathbb {R}$ using $\mathbb {N}_{\varpi }=\big \{\varpi ,\varpi +1,\varpi +2,...\big \},$ described as

2.1 $$\begin{aligned} \,^{c}\Delta _{\varpi }^{\beta }\digamma (\xi )= & {} \Delta _{\varpi }^{-(\mathfrak {n}-\beta )}\Delta ^{\mathfrak {n}}\digamma (\xi )\nonumber \\= & {} \frac{1}{\Gamma (\mathfrak {n}-\beta )}\sum \limits _{\textbf{u}=\varpi }^{\xi -(\mathfrak {n}-\beta )}(\xi -\textbf{u}-1)^{(\mathfrak {n}-\beta -1)}\Delta ^{\mathfrak {n}}\digamma (\textbf{u}), \end{aligned}$$

where $\beta \in \mathbb {N}$ is the FO, $\xi \in \mathbb {N}_{\varpi +\mathfrak {n}-\beta }$ and $\mathfrak {n}=\lceil \beta \rceil +1.$ The $\beta ^{th}$ fractional sum of $\Delta _{\textbf{u}}^{\mathfrak {n}}\digamma (\xi )$ in (2.1) is described analogously to^40,41^ as

2.2 $$\begin{aligned} \Delta _{\varpi }^{-\beta }\digamma (\xi )= \frac{1}{\Gamma (\beta )}\sum \limits _{\textbf{u}=0}^{\xi =\beta }(\xi -\textbf{u}-1)^{(\beta -1)}\digamma (\textbf{u}), \end{aligned}$$

alongside $\xi \in \mathbb {N}_{\varpi +\beta },~\beta >0$. The falling factor $\xi ^{(\beta )}$ established as a consequence of the Gamma function is denoted by the symbol $\Gamma$ as

2.3 $$\begin{aligned} \xi ^{(\beta )}=\frac{\Gamma (\xi +1)}{\Gamma (\xi +1-\beta )}. \end{aligned}$$

The results that adhere serve as a framework for the computational approach and stability evaluation that we must perform throughout the research whenever interacting with the suggested DFO mechanisms.

### Theorem 2.1

Reference^42^

Assume that there is a FO difference formula

2.4 $$\begin{aligned} {\left\{ \begin{array}{ll} \,^{c}\Delta _{\varpi }^{\beta }\chi (\xi )=\Theta \big (\xi +\beta -1,\chi (\xi +\beta -1)\big ),\\ \Delta ^{\wp }\chi (\varpi )={\chi }_{\wp },~~\mathfrak {n}=\lceil \beta \rceil +1,~~\wp =0,1,...,\mathfrak {n}-1 \end{array}\right. } \end{aligned}$$

the corresponding discrete integral the formula is as follows:

2.5 $$\begin{aligned} \chi (\xi )={\chi }_{0}(\xi )+\frac{1}{\Gamma (\beta )}\sum \limits _{\textbf{u}=\varpi +\mathfrak {n}-\beta }^{\xi -\beta }(\xi -\textbf{u}-1)^{(\beta -1)}\Theta \big (\textbf{u}+\beta -1,\chi (\textbf{u}+\beta -1)\big ),~~\xi \in \mathbb {N}_{\varpi +\mathfrak {n}}, \end{aligned}$$

where

2.6 $$\begin{aligned} {\chi }_{0}(\xi )=\sum \limits _{\wp =0}^{\mathfrak {n}-1}\frac{(\xi -\varpi )^{(\wp )}}{\Gamma (\wp +1)}\Delta ^{\wp }\chi (\varpi ). \end{aligned}$$

### Theorem 2.2

Reference^43^

Assume that the unsteady state of the linear DFO framework

2.7 $$\begin{aligned} \,^{c}\Delta _{\varpi }^{\beta }\digamma (\xi )=\mho \digamma (\xi +\beta -1), \end{aligned}$$

where $\digamma (\xi )=\big (\digamma _{1}(\xi ),...,\digamma _{\mathfrak {n}}(\xi )\big )^{\textbf{T}},~\beta \in (0,1],~~\mho \in \mathbb {R}^{\mathfrak {n}\times \mathfrak {n}}$ and for all $\xi \in \mathbb {N}_{\varpi +1-\beta }$ is asymptotically stable if

2.8 $$\begin{aligned} \varsigma \in \Big \{\vartheta \in \mathbb {C}:\vert \vartheta \vert <\Big (2\cos \frac{\vert \arg \vartheta -\pi \vert }{2-\beta }\Big )^{\beta }~and~\vert \arg \vartheta \vert >\beta \pi /2\Big \} \end{aligned}$$

$\forall$ the eigenvalues $\varsigma$ of $\mho .$

## INN model and its FO formulation

The electric-power interactions associated with a specific capacitance were calculated as $\mathcal {C}\frac{d\mathcal {U}^{\beta }}{d\xi ^{\beta }}=\Im ,$ where $\mathcal {U},~\mathcal {C}$ and $\mathcal {R}$ denote the electrostatic electric current, cellular capacitors and cell obstruction whiles $\beta \in (0,1)$ is the fractional factor. The FO differential formulation^10^ is able to be implemented for determining the FO structure of the inactive cell electrical energy relationship. Previous research demonstrated that a FO conductive concept might be suitable for describing and investigating the functioning of inactive cell patterns^44^. Furthermore, fractional-order interactions are applicable to specify long-term memory implications attributed to neural plasticity and specific cell stimulation, insulator impact, and radioactive implications^44^. In the present research, we anticipate the DFO interactions of the Izhikevich system^8,45^ and show how cell power at different commensurate and incommensurate affects the features of NNs over numerous time frames. Initially, we supply an executive summary regarding the DFO Izhikevich simulation and describe the neurological features of cellular rises. We investigate the barely noticeable fluctuations and surge development that characterize launching procedures. In conclusion, we look at the properties of an ensemble of DFO NNs.

In 2003, Izhikevich^8^ contemplated an INN that is capable of multiple kinds of cortical-in-nature neuronal cell spikes and collapses. It makes neurological sense as HH patterns while being practically productive as integrate-and-fire neurons in general. The continuous-time FO Izhikevich approach, which relies on the classical Izhikevich framework, is illustrated by a couple of system parameters $\textbf{x}(t_1)$ and $\textbf{y}(\xi )$ as follows:

3.1 $$\begin{aligned} {\left\{ \begin{array}{ll} \textbf{D}^{\beta }\textbf{x}(\xi )=0.04\textbf{x}^{2}-\textbf{y}+5\textbf{x}+140+\Im ,\\ \textbf{D}^{\beta }\textbf{y}(\xi )=\sigma (\eta \textbf{x}-\textbf{y}),\end{array}\right. } \end{aligned}$$

where the FO residing in the range $\beta \in (0,1)$. Take into account a framework via proportional to FO. At $\beta =1$, the framework diminishes to the classical Izhikevich approach. The membrane power is represented by the structure’s component $\textbf{x}$, and the reactivation component $\textbf{y}$ determines the stimulation of $\mathcal {K}^{+}$ and suppression of ${Na}^{+}$ electrostatic berries. The bursting trends are modulated by FO fluctuations in electrostatic flows^46^. When the cellular power attains maximum numbers, $\textbf{x}_{\ell \wp }$, both of the components listed below evolve into

3.2 $$\begin{aligned} ~\textbf{x}_{\ell \wp }\le \textbf{x}\implies ~~{\left\{ \begin{array}{ll} \textbf{x}\leftarrow \psi ,\\ \textbf{y}\leftarrow \textbf{y}+\nu . \end{array}\right. } \end{aligned}$$

At this point, $\textbf{x}_{\ell \wp }=30(\mathfrak {m}\mathcal {U})$ is implemented. Also, $\sigma ,~\eta ,~\psi$ and $\nu$ are devoid of dimension variables. The equilibrium possibilities are between 70 and 60 $\mathfrak {m}\mathcal {U}$, depending on the value of $\eta$. The value of $\sigma$ denotes the duration of the restoration factor, $\textbf{y}$. The value of $\eta$ represents the responsiveness of the recuperating mechanism factor $\textbf{y}$ to barely noticeable oscillations in the cellular power, $\textbf{x}$. The data points $\psi$ and $\nu$ represent the after spike restored values of $\textbf{x}$ and $\textbf{y}$ resulting from promptly high-threshold $\mathcal {K}^{+}$ transmit insulators and reluctantly high-threshold ${Na}^{+}$ and $\mathcal {K}^{+}$ insulators, as well as various appropriate setting selections that influence multiple kinds of launching structures that frequently appear in neocortical^47^ and thalamic neuronal cells^48^. The differences in setting are taken into account as described in the studies^8,49^.

The initial values are taken to be $\textbf{x}=-63$ and $\textbf{y}=\eta \textbf{x}$^8,49^. It should be noted that simply by differing in such undefined settings, distinctive launching features of traditional Izhikevich nerve cells (that is, consistently exploding, chattering and exploding) could be accomplished. Multiple varieties of spikes and overflowing variations are frequently identified in neocortical cells in neurological systems for inside cells files^47,48^, as well as excitement neural activity by Izhikevich^8,49^. We evaluate an identical strategy of spike-bursting procedures for several DFOs.

## Qualitative analysis of DFO-INN system

In this section, the behaviour of the DF-INN framework (3.1) via cortical neurons will be investigated in the following situations: commensurate order, incommensurate order and VO. These tests will be carried out employing a variety of numerical modelling techniques, including exhibit phase profiles, bifurcation schematics, and maximum Lyapunov exponent ($\zeta _{\max }$) predictions. The Jacobian matrix strategy^50^ will be used to figure out the $\zeta _{\max }$ of the attracted components of the DF-INN framework (3.1).

### Commensurate DFO-INN system

In this subsection, we are going to study the evolution of the DFO-INN framework. We will go over the features of the suggested commensurate DF-INN framework (4.1). It deserves to be taken into account that a collection of formulae with commensurate order is a set of formulae obtained via similar inquiries. Given that, we shall subsequently offer a quantifiable equation generated by Theorem 2.1 in the following manner:

4.1 $$\begin{aligned} {\left\{ \begin{array}{ll} \textbf{x}(\mathfrak {n}+1)=0.04\textbf{x}^{2}(\mathfrak {n})-\textbf{y}(\mathfrak {n})+5\textbf{x}(\mathfrak {n})+140+\Im ,\\ \textbf{y}(\mathfrak {n}+1)=\sigma \big (\eta \textbf{x}(\mathfrak {n})-\textbf{y}(\mathfrak {n})\big ), \end{array}\right. } \end{aligned}$$

where $\textbf{x}(\mathfrak {n})$ and $\textbf{y}(\mathfrak {n})$ are the system’s indications and have certain factors $\sigma ,~\eta ,~\psi$ and $\nu$. Considering the system information in two data sets:

Set ($$B_{1}$$) $$(\sigma ,\eta ,\psi ,\nu )=(0.2,2,-55,4)$$ and $$\Im \ge 3$$,

Set ($B_{2}$) $(\sigma ,\eta ,\psi ,\nu )=(0.02,0.2,-56,-16)$ and $\Im \ge -105$. It has been demonstrated that the DFO-INN system (4.1) has chaotic patterns. The first-order difference of the DFO-INN system, (4.1) can be expressed as

4.2 $$\begin{aligned} {\left\{ \begin{array}{ll} \Delta \textbf{x}(\mathfrak {n})=0.04\textbf{x}^{2}(\mathfrak {n})-\textbf{y}(\mathfrak {n})+5\textbf{x}(\mathfrak {n})+140+\Im -\textbf{x}(\mathfrak {n}),\\ \Delta \textbf{y}(\mathfrak {n})=\sigma \big (\eta \textbf{x}(\mathfrak {n})-\textbf{y}(\mathfrak {n})\big )-\textbf{y}(\mathfrak {n}). \end{array}\right. } \end{aligned}$$

The DFO-INN model (4.3) tends to be obtained by employing the Caputo-like delta difference described in (2.1) which serves as the initial value problem. The fractional difference form of (3.1) is

4.3 $$\begin{aligned} {\left\{ \begin{array}{ll} \,^{c}\Delta _{\varpi }^{\beta }\textbf{x}(\xi )=0.04\textbf{x}^{2}(\xi -1+\beta )-\textbf{y}(\xi -1+\beta )+5\textbf{x}(\xi -1+\beta )+140+\Im -\textbf{x}(\xi -1+\beta ),\\ \,^{c}\Delta _{\varpi }^{\beta }\textbf{y}(\mathfrak {n})=\sigma \big (\eta \textbf{x}(\xi -1+\beta )-\textbf{y}(\xi -1+\beta )\big )-\textbf{y}(\xi -1+\beta ), \end{array}\right. } \end{aligned}$$

for $\beta \in (0,1]$ and $\xi \in \mathbb {N}_{\varpi +1-\beta }.$ It is worth noting that the FOs of both fractional differences in (4.3) are alike, resulting in the phenomenon known as a commensurate mechanism.

In view of Theorem 2.1, we find

4.4 $$\begin{aligned} {\left\{ \begin{array}{ll} \textbf{x}(\xi )=\textbf{x}(\varpi )+\frac{1}{\Gamma (\beta )}\sum \limits _{\textbf{u}=\varpi }^{\xi -\beta }(\xi -\textbf{u}-1)^{(\beta -1)}\Big (0.04\textbf{x}^{2}(\xi -1+\beta )-\textbf{y}(\xi -1+\beta )+5\textbf{x}(\xi -1+\beta )+140+\Im \\ \qquad \qquad -\textbf{x}(\xi -1+\beta )\Big ),\\ \textbf{y}(\xi )=\textbf{y}(\varpi )+\frac{1}{\Gamma (\beta )}\sum \limits _{\textbf{u}=\varpi }^{\xi -\beta }(\xi -\textbf{u}-1)^{(\beta -1)}\Big (\sigma \big (\eta \textbf{x}(\xi -1+\beta )-\textbf{y}(\xi -1+\beta )\big )-\textbf{y}(\xi -1+\beta )\Big ), \end{array}\right. } \end{aligned}$$

where $\frac{(\xi -\textbf{u}-1)^{(\beta -1)}}{\Gamma (\beta )}$ symbolizes the discrete kernel, which is defined as

4.5 $$\begin{aligned} \frac{(\xi -\textbf{u}-1)^{(\beta -1)}}{\Gamma (\beta )}=\frac{\Gamma (\xi -\textbf{u})}{\Gamma (\beta )\Gamma (\xi -\textbf{u}-\beta +1)} \end{aligned}$$

and for $\varpi =0$ produce the following scheme

4.6 $$\begin{aligned} {\left\{ \begin{array}{ll} \textbf{x}(\mathfrak {n})=\textbf{x}(0)+\frac{1}{\Gamma (\beta )}\sum \limits _{\jmath =1}^{\mathfrak {n}}\frac{\Gamma (\mathfrak {n}-\jmath +\beta )}{\Gamma (\mathfrak {n}-\jmath +1)}\Big (0.04\textbf{x}^{2}(\jmath -1)-\textbf{y}(\jmath -1)+5\textbf{x}(\jmath -1)+140+\Im -\textbf{x}(\jmath -1)\Big ),\\ \textbf{y}(\mathfrak {n})=\textbf{y}(0)+\frac{1}{\Gamma (\beta )}\sum \limits _{\jmath =1}^{\mathfrak {n}}\frac{\Gamma (\mathfrak {n}-\jmath +\beta )}{\Gamma (\mathfrak {n}-\jmath +1)}\Big (\sigma \big (\eta \textbf{x}(\jmath -1)-\textbf{y}(\jmath -1)\big )-\textbf{y}(\jmath -1)\Big ). \end{array}\right. } \end{aligned}$$

In order to assess the framework’s stablization, we need to identify the fixed points $(\textbf{x}^{*},\textbf{y}^{*})$. For this, we can do by comparing the right-hand side equal to 0, resulting in $\eta \textbf{x}(\jmath -1)=\textbf{y}(\jmath -1)$ and $0.04\textbf{x}^{2}(\jmath -1)-\textbf{y}(\jmath -1)+5\textbf{x}(\jmath -1)+140+\Im =0$. Assume that $\mathcal {E}=(\textbf{x}^{*},\textbf{y}^{*})$ is the fixed point, then the Jacobian matrix at $\mathcal {E}$ can be expressed as

4.7 $$\begin{aligned} \mathcal {J}=\begin{pmatrix} 0.08\textbf{x}^{*}+5&{}-1\\ \sigma \eta &{}-\sigma \end{pmatrix}. \end{aligned}$$

Take a glance at the parameterized set ($B_{1}$) having $\Im =10.$ Moreover, $\mathcal {E}_{1}=-60.0\pm 12.2474489\iota ,~\mathcal {E}_{2}=-12.0\pm 2.4495\iota ,$ the eigenvalues that represent the two fixed points are $\varsigma _{1}=0.19910+0.9837\iota$ and $\varsigma _{2}=-0.0191-0.0039\iota$ in regard to $\mathcal {E}_{1}$ and $\varsigma _{1},\varsigma _{2}$ via appreciation to $\mathcal {E}_{2}$. In such a particular instance, the steady states are asymptotically stable if they fulfill $\beta <\frac{2}{\pi }\min \limits _{\iota }\big \vert \arg (\varsigma _{\iota })\big \vert \approx (2.7422/\pi )\approx 0.8730.$ The framework has a pair of real steady states $\mathcal {E}_{1}=(-17.89999,-36.0001)$ and $\mathcal {E}_{1}=(-57.00467,~-115.0001)$ using $\Im =98$ at component establish in set ($B_{2}$). The eigenvalues that represent both fixed points are $\varsigma _{1}=3.4657$ and $\varsigma _{2}=-0.0908$ for $\mathcal {E}_{1}$ signifies the saddle node and $\varsigma _{1},\varsigma _{2}=0.11990\pm 0.54689\iota$ for $\mathcal {E}_{2}$ (that is, an unsteady concentrate), indicating that it is asymptotically steady when $\beta <\frac{2}{\pi }\min \limits _{\iota }\big \vert \arg (\varsigma _{\iota })\big \vert \approx 0.8656.$ The computational findings at setting set ($B_{2}$) confirm the aforementioned stabilization the requirements of the actual stable state approach to $\mathcal {E}_{2}$.

As previously stated, DFC incorporates the significant benefit of infinite collective memory. This is readily apparent in (4.7), in which the outcome $\textbf{x}(\mathfrak {n})$ is dependent on all preceding information $\textbf{x}(0),...,\textbf{x}(\mathfrak {n}-1).$ Obviously, this is not the situation regarding the classical sense of framework (4.1). Utilizing the numerical data (4.7), a Matlab activity was developed.

**Figure 1:**
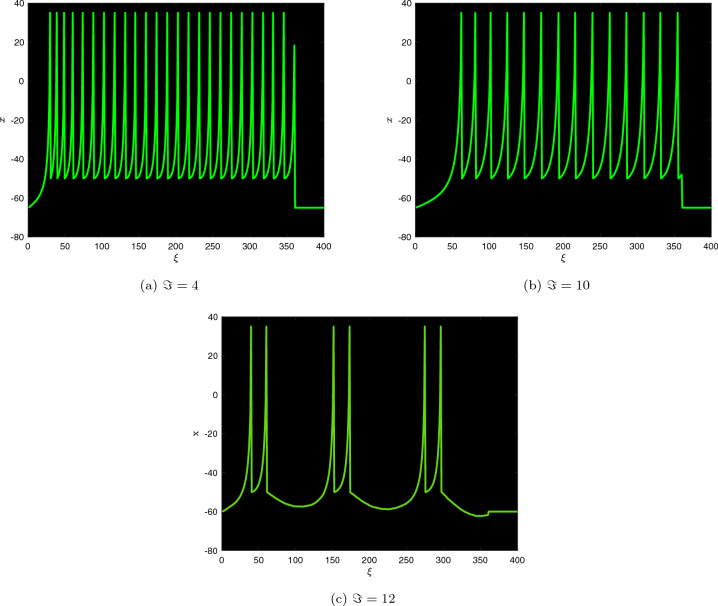
The commensurate DFO-INN system (4.1) generates (**a**) tonic spiking pattern, (**b**) bursting pattern (**c**) chattering behaviour when $\beta =0.90$.

**Figure 2:**
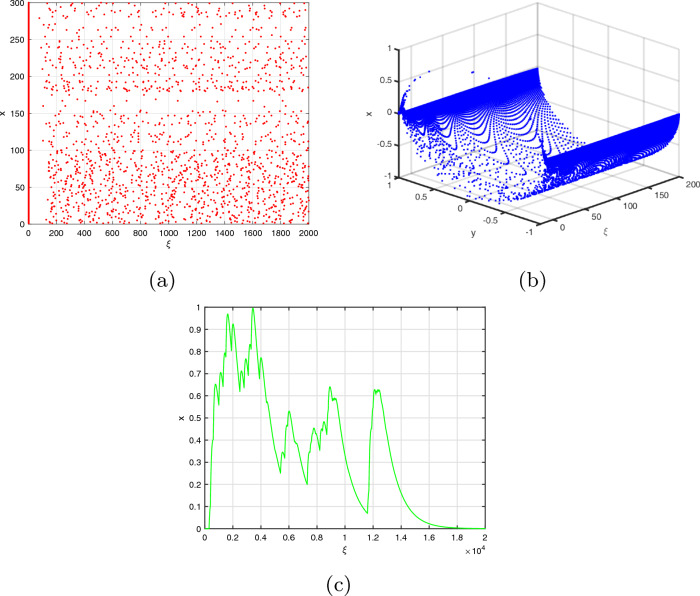
The commensurate DFO-INN system (4.7) generates the NN actions for the set ($B_{1}$), when $\beta =0.90$ and $\Im <4$ in this case the neurons in the network do not produce any spiking activity.

**Figure 3:**
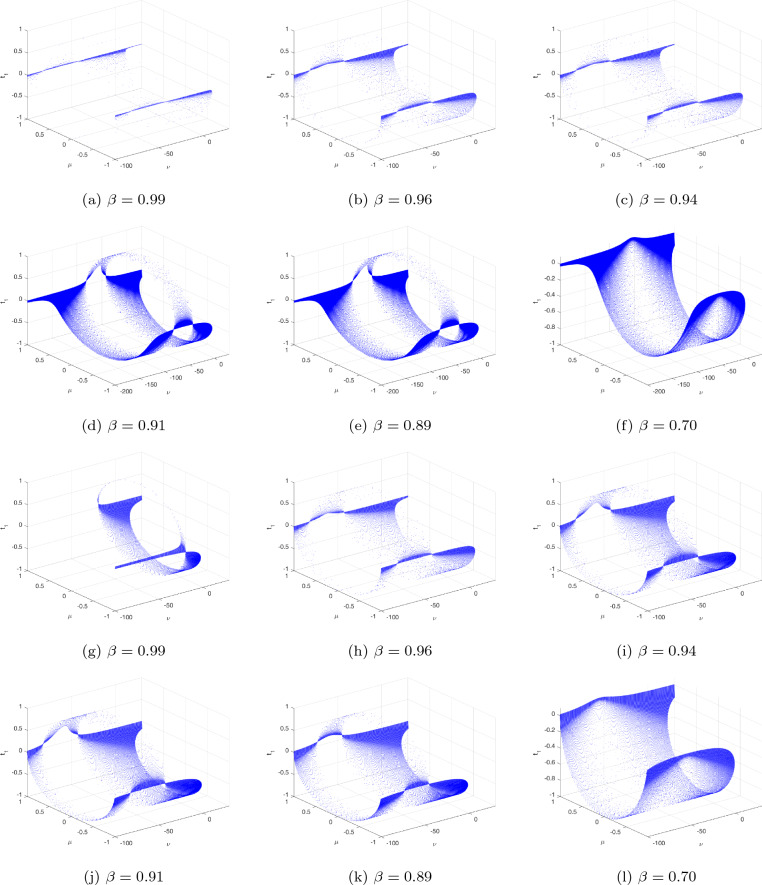
Phase illustrations of the commensurate DFO-INN system (4.7) generate various kinds of spikes in, inherently overflowing chattering behaviour for different FOs, including system parameters set $B_{1}$.

We can calculate the neuron activities of the commensurate DFO-INN (4.1) model for $\beta =0.9$ by displaying the result $(\textbf{x}(\mathfrak {n}),\textbf{y}(\mathfrak {n}))$ in the $\textbf{x}-\textbf{y}$ plane, as shown in Fig. 1. The ICs $(\textbf{x}(0),\textbf{y}(0))$^8,49^ and bifurcation factors were determined for $\Im <4.$ The bifurcation visualization incorporating a crucial value is shown in Fig. 2a,b and the $\zeta _{\max }$ as a function determined by applying the Jacobian methodology is shown in Fig. 2c. These scenarios affirm the presence of chaos and reinforce prior findings in the available research. When the energy stimulation is $\Im <4$, the commensurate DFO INN system exhibits no spikes in or brimming behaviour at the setting that initiates ($B_{1}$), At $\Im =3,$ the steady stats are $(\textbf{x}^{*},\textbf{y}^{*})=(-65,-13)$ and (55, 11). The associated eigenvalues are **(i)** $\varsigma _{1},\varsigma _{2}=(-0.1740,-0.0460)$ and (0.5935, 0.0135), respectively. The primary erroneously neutral state approach in the aforementioned setting is a steady node and the next one is a saddle point, that is, unsteady. At this point, suppose $\Im =4,$ and there is a single fixed point $(\textbf{x}^{*},\textbf{y}^{*})=(-60,-12)$ with associated eigenvalues $\varsigma _{1},\varsigma _{2}=(0.18, 0).$ In the following, we concentrate on ICs and Set ($B_{1}$) and vary the DFO in the range (0, 1). We developed the DFO-INN model for 6000 points and calculated the outcome $(\textbf{x}(\mathfrak {n}),\textbf{y}(\mathfrak {n}))$ in the $\textbf{x}-\textbf{y}$ plane for the FOs 0.99,  0.96, 0.94, 0.91, 0.89 and 0.70. As shown in Fig. 3, the enticement changes as frequently as the FO changes. In the context of every scenario, the process space settles on a restricted attractant. We observe that as being $\beta$ falls, the outcome addresses a certain amount of highlights as long as the smallest amount of variations ${\mathfrak {n}}_{0}$ thereafter frequently deviates infinitely. As an illustration, ${\mathfrak {n}}_{0}=1854$ when $\beta =0.70$ (see Figure 3(**f**)). The mathematical results shown in Fig. 3a–k show that the computed result $(\textbf{x}(\mathfrak {n}),\textbf{y}(\mathfrak {n}))$ is dependent upon the FO.

**Figure 4:**
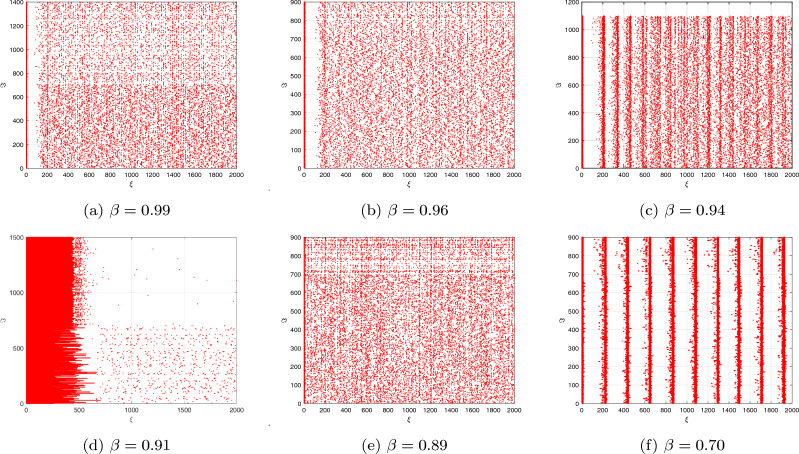
The NN response of the DFO-INN model (4.7) for various DFO with a set of parameters stated under assumptions ($B_{1}$) and current stimuli $\Im ;$ (**a**) no spiking; (**b**) small spiking; (**c**) the network started producing cortical-like asynchronous dynamics; (**d**) firing activity pattern; )**e**) synchronized firings disappear; (**f**) synchronized firings.

Furthermore, we employ bifurcation illustrations that include the significant factor to learn additional information regarding the behaviour of the DFO-INN system (4.7). We fluctuate in measures of $\Delta \xi =0.005$ across the range [0, 2] and pick ICs according to^8,49^ and $\Im =3.5.$ Fig. 4 depicts the bifurcation schematics for 0.99,  0.96, 0.94, 0.91, 0.89 and 0.70. When $\beta =1$, the DFO-INN (4.7) demonstrates a changing pattern depicted in Fig. 1a, that, appropriately, corresponds to the normative bifurcation lead described in the scientific literature. The map connects to an individual fixed point in the interval $3<\Im <4$ Then, as $3.5<\Im \le 4.5$, non-hyperbolic equilibrium methods are inherently unstable. Compact fluctuations may result in a specific bifurcation linked with the non-hyperbolic states, which can lead to the phenomenon fluctuating from rigidity, vanishing, or being separated from numerous fixed points. Whenever the electrical power stimulus data, $\Im$, raises from 3 to 4, the two steady states proceed towards the others, interact, and annihilate. It experiences a saddle node bifurcation, in which a junction point and a stable component address adjacent ones, merge into an isolated fixed point, and then disintegrate as $\Im >4$ and it operates in every FO to a completely constructed chaotic system, as shown in Fig. 4. The explosion variations of the DFO-INN model with various FOs, as well as the classical case interactions for $\Im \ge 4$, are investigated.

As demonstrated by Fig. 4, the FO influences the bifurcation plot’s broadening transform in addition to the time frame of the erratic region. The bifurcation illustration for $\beta =0.96$ corresponds to the pertinent numerical illustration, with the exception of an insignificant improvement in the range in which chaotic behaviour is noticed. Now, the DFO-INN system (4.7) generates a variety of bursting procedures based on FO modifications at constant electrical stimulation. Thanks to a preset inserted current $\Im =4$, the DFO-INN system generates instinctively exploding at $\beta = 0.94$, chattering at $\beta =0.89$ and regularly exploding at $\beta =0.70$ in deeper interspike duration (see Figure 4). When we minimize the DFOs more significantly, the explosion time frame expands, resulting in spiked oscillations such as ( for $\beta =0.99$, it generates (a) no spiking; for $\beta =0.96$, it generates (b) small spiking; for $\beta =0.94$, it generates (c) the network started producing cortical-like asynchronous dynamics; for $\beta =0.91$, it generates (d) firing activity pattern; for $\beta =0.89$, it generates (e) synchronized firings disappear; for $\beta =0.70$, it generates (f) synchronized firings, respectively).

**Figure 5:**
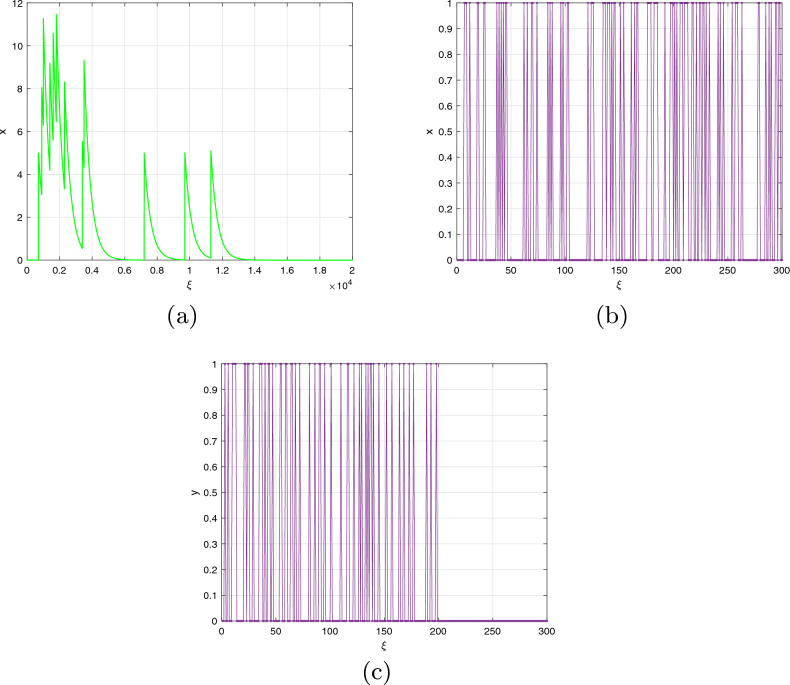
The $\zeta _{\max }$ response of the DFO-INN model (4.7) for DFO $\beta =0.5$ with a set of parameters stated under assumptions ($B_{1}$) and current stimuli $\Im =12.$.

As we lessen $\beta$ (while the other factors remain constant), we identify that DFO-INN system generates muttering at $\beta =0.99$ and subsequently deviates out of the integer form framework that uses a stable current stimulation $\Im =10$, the orbit no longer passes to a fixed point. Indeed, as $\mathfrak {n}$ rises, the pattern of motion turns limitless (see Fig. 5). The range within which chaos can be detected differs significantly within the bifurcation diagrams of the classical and DFO-INN systems. Therefore, the FO model results in distinctive fluctuations. When the fractional order is reduced to 0.95, it generates hybrid form fluctuations.

As $\beta$ diminishes, the time frame appears a bit shorter. The $\zeta _{\max }$ of the DFO-INN derived from the fractional Jacobian procedure described in^50^ is shown in Figure 5 (**a**). The following diagram was produced employing the identical former factors and ICs as before, including $\beta =0.99$ and ordinary exploding. In this case, the DFO-INN model generates deeper brimming via a further exploded time frame. The stimulation structure shifts to more prolonged exploding, with a boost throughout both the stage of activity (that is, promptly exploding and bursting) as well as the inactive stage. The outcome is perfectly consistent regarding the analogous bifurcation layout. Furthermore, as the FO decreases, the oscillatory trends transform concerning exploding to swiftly spikes in at $\beta =0.5$ (see Fig. 5b,c). Through the energy stimulation $\Im =12$, the DFO-INN framework controls from chattering to overflowing as FOs decline, producing deeper exploding regarding more frequently inter-spike in the specified time frame and then swiftly spiked as FOs minimize more deeply. Figure 1 depicts the contends of the DFO-INN with 3000 iterations when set $B_{1}$ and $\beta =0.90$ are assumed.

### Noncommensurate DFO-INN system

The behaviour of the FO-INN model with non-commensurate FO parameters is investigated in this subsection. The practise of employing distinguished FOs for every formula of the framework is referred to as the non-commensurate order system. The representation of the non-commensurate DFO-INN can be viewed as

4.8 $$\begin{aligned} {\left\{ \begin{array}{ll} \,^{c}\Delta _{\varpi }^{\beta _{1}}\textbf{x}(\xi )=0.04\textbf{x}^{2}(\textbf{r}-1+\beta _{1})-\textbf{y}(\textbf{r}-1+\beta _{1})+5\textbf{x}(\textbf{r}-1+\beta _{1})+140+\Im ,~~\textbf{r}\in \mathbb {N}_{\varpi -\beta _{1}+1}\\ \,^{c}\Delta _{\varpi }^{\beta _{2}}\textbf{y}(\mathfrak {n})=\sigma \big (\eta \textbf{x}(\textbf{r}-1+\beta _{2})-\textbf{y}(\textbf{r}-1+\beta _{2})\big ),~~\textbf{r}\in \mathbb {N}_{\varpi -\beta _{2}+1}. \end{array}\right. } \end{aligned}$$

The quantitative framework of the incommensurate DFO-INN system (4.8) can be written according to the Theorem 2.1:

4.9 $$\begin{aligned} {\left\{ \begin{array}{ll} \textbf{x}(\textbf{r}+1)=\textbf{x}(0)+\sum \limits _{\mathfrak {n}=0}^{\wp }\frac{\Gamma (\textbf{r}-1-\wp +\beta _{1})}{\Gamma (\beta _{1})\Gamma (\xi -\wp )}\Big (0.04\textbf{x}^{2}(\wp )-\textbf{y}(\wp )+5\textbf{x}(\wp )+140+\Im \Big ),\\ \textbf{y}(\textbf{r}+1)=\textbf{y}(0)+\sum \limits _{\mathfrak {n}=0}^{\wp }\frac{\Gamma (\textbf{r}-1-\wp +\beta _{2})}{\Gamma (\beta _{2})\Gamma (\xi -\wp )}\Big (\sigma \big (\eta \textbf{x}(\wp )-\textbf{y}(\wp )\big )\Big ),~~\textbf{r}=1,2,...~. \end{array}\right. } \end{aligned}$$

Currently, we examine the settings set ($B_{2}$), which has different inserted energy stimulation, $\Im$, when the structure’s inherited factors in the context of Andronov-Hopf bifurcation produce a restriction process from a steady state solution in a self-governing evolving technique whenever the steady state modifies its degree of stability via the combination of entirely fictitious eigenvalues. These representations are clearly distinct, implying that changes in FOs $\beta _{1}$ and $\beta _{2}$ have an effect on the statuses of the incommensurate DFO-INN system (4.9). It denotes the immediate conception or demise of a recurring approach coming from equilibrium when a system’s prevailing value traverses a critical threshold. As a result, a bifurcating Hopf is feasible and appears in mechanisms via a scale greater than or equal to two. Take into account the DFO-INN system (4.9) containing the prevalent setting $\Im$, where the state of balance point $\mathcal {E}=(\textbf{x}^{*},\textbf{y}^{*})$ is dependent on $\Im$. Assume the Jacobian matrix’s eigenvalues, $\mathcal {J}$, with respect to the fixed point $\mathcal {E}$ become $\varsigma (\Im ),\tilde{\varsigma }(\Im )=\phi _{1}(\Im )\pm \iota \phi _{2}(\Im )$. Assume that the subsequent influences have been fulfilled for a specific significant level $\Im$, clarify that $\Im =\Im _{0}.$ For example, for $(\beta _{1},\beta _{2})=(1,0.9)$, we have evidence that the structure’s contends transform from erratic to recurring as the energy estimation $\Im$ increases. The chaotic region is apparent for all $(\beta _{1},\beta _{2})=(0.9,0.3)$, excluding a restricted area when $\Im$ nears 10, whereas for $(\beta _{1},\beta _{2})=(0.5,1)$, when the value of $\Im$ improves and towards $\Im =-104$, the incommensurate DFO-INN system (4.9) demonstrates regular regions alongside oscillatory circular orbits. In addition, we examine the two additional situations to provide an improved illustrative of the impact of incommensurate DFO-INN system’s practises (4.9):

($$A_{1}$$):
 At the significant threshold of $\Im$ adjacent to the equilibrium point $\mathcal {E}$, the matrix $\mathcal {J}$ possesses a straightforward set of entirely fictitious eigenvalues, which shows that at $\Im =\Im _{0},$ $\phi _{1}=0$ and $\phi _{2}=\omega \ne 0$ referred to constitute the non-hyperbolicity criteria. Then the result is a uniform spectrum using a steady state at the threshold of $\Im$ and exclusively fictional eigenvalues that fluctuate efficiently as $\Im$ changes.
($$A_{2}$$):
 When $\frac{d\phi _{1}(\Im )}{d\Im }\Big \vert _{\Im =\Im _{0}}=\nu \ne 0$ referred to for being the transversality state to the network endures a Hopf bifurcation.

**Figure 6:**
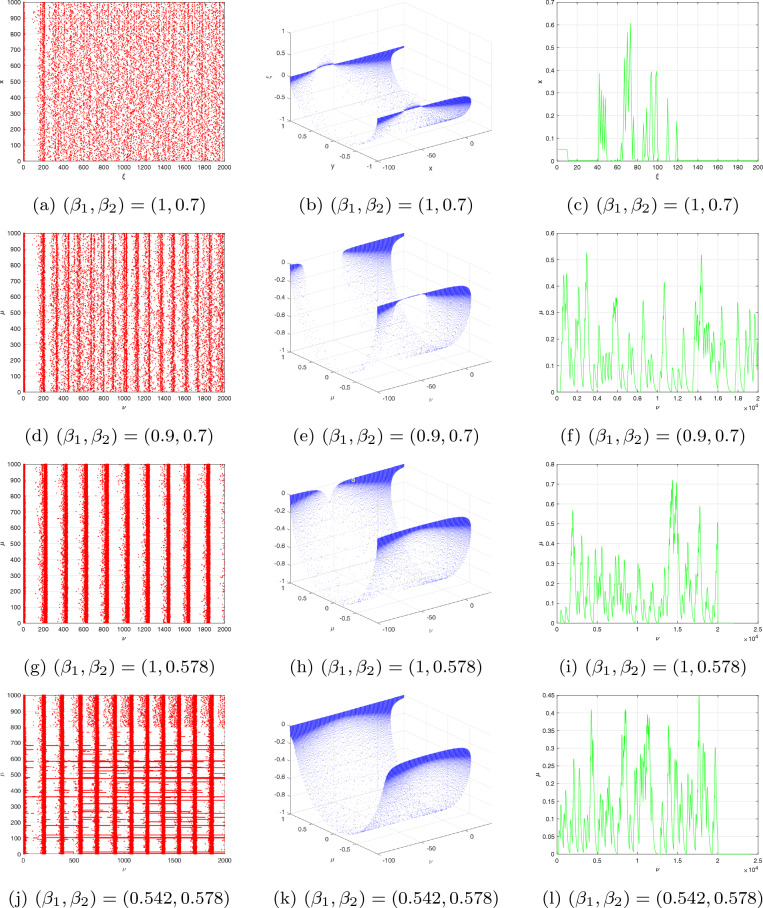
Bifurcation, chaos and $\zeta _{\max }$ behaviour of the neural activities for non-commensurate DFO-INN system (4.9) generate various kinds of spiking and bursting patterns for various current stimuli $\Im$ using parametric sets ($B_{1}$) and ($B_{2}$) such as (**a**) small spiking; (**b**) synchronized firings; the network started producing cortical-like asynchronous dynamics; (**c**) occasional events of synchronized firings, respectively.

Consider that the characteristic polynomial has two exclusively complex factors for applying the Hopf bifurcation assessment to the evaluation. The steady state solution is the result that includes the formulations $\textbf{y}=\eta \textbf{x}$ and $0.04\textbf{x}^{2}+(5-\eta )\textbf{x}+140+\Im =0.$ Then the steady state is $\textbf{x}^{*}=-3\pm \sqrt{-0.16\Im -13.4}/0.08$ and $\textbf{y}^{*}=2\textbf{x}^{*}.$ Thus, the characteristic equation reduces to $g_{1}(\varsigma ,\Im )=\varsigma ^{2}+(\sigma -0.08\textbf{x}^{*}-5)\varsigma +(\sigma \eta -5\sigma -0.08\sigma \textbf{x}^{*})=0;$ after plugging the values of $\sigma$ and $\eta ,$ we have $g_{1}(\varsigma ,\Im )=\varsigma ^{2}+(4.8+0,08\textbf{x}^{*})\varsigma -(0.016\textbf{x}^{*}+0.6)=0$ and $\varsigma (\Im ),\tilde{\varsigma }(\Im )=\phi _{1}(\Im )\pm \iota \phi _{2}(\Im ),$ where $\phi _{1}(\Im )=-0.5(\sigma -0.08\textbf{x}^{*}-5)$ and $\phi _{2}(\Im )=0.5\sqrt{(\sigma -0.08\textbf{x}^{*}-5)^{2}+4\sigma \eta +20\sigma +0.32\sigma \textbf{x}^{*}}.$ We change FO $\beta _{1}$ from 0 to 1 using an increment size of $\Delta \beta _{1}=0.005$. Figures 6(**a**-**c**) demonstrate the bifurcation and their associated $\zeta _{\max }$ for $(\beta _{1},\beta _{2})=(1,0.7)$ with $\Im =\Im _{0}=-100.$ According to Fig. 6, the configuration of the incommensurate DFO-INN (4.9) exhibits chaos behaviour for lesser determines of $\beta _{1}$, as confirmed by $\zeta _{\max }$, as shown in Fig. 6(**b**). For the DFO $\beta _{1}=1$, the $\zeta _{\max }$ illustrated in Fig. 6c alternates between the two extremes. The findings indicate the emergence of a chaotic region with recurring views.

To demonstrate the bifurcation requirement, we must determine $\Im =\Im _{0}$, which corresponds to the significant threshold of bifurcation and is capable of being generated whenever $\hbar (\Im )=4.8+0.08\textbf{x}^{*}=0$ employing the opposite sign of the steady state $\textbf{x}^{*}$. The quantity is changed to $\Im =\Im _{0}=-103$, which means that at that level, the resulting differentiation of $\phi _{1}(\Im )$ and the steady factor of $g_{1}(\varsigma ,\Im _{0})$ are nonzero. The structure encompasses a pair of exclusively imaginary eigenvalues at this point in time. Thanks to $\Im =107$, the steady state $(\textbf{x}^{*},\textbf{y}^{*})=(62.64358,124.78821)$ is a steady prioritize because the eigenvalues are $\varsigma _{1},\varsigma _{2}=(-0.02321\pm 0.61269\iota )$. The newly generated equilibrium approach $(\textbf{x}^{*},\textbf{y}^{*})=(-63,-124)$ via $\Im =105$ includes exclusively fictitious eigenvalues $\varsigma _{1},\varsigma _{2}=\pm 0.6731\iota$. The equilibrium approach turns unpredictable when we improve the electrical stimulation $\Im =-104.$ A bifurcation happens when a stable outcome eliminates its rigidity when the intricate conjugate eigenvalues traverse the multifaceted plane’s fictitious axes. The bifurcation and its $\zeta _{\max }$ are depicted in Fig. 6d–f to investigate the fluctuating behaviours of the incommensurate DFO-INN (4.9) when $\beta _{2}$ is a configurable FO. These outcomes can be achieved by differing $\beta _{2}$ in the interval (0, 1] and via incommensurate FOs $\beta _{1}=0.9.$ We comprehend that whenever the $\beta _{2}$ is inadequate, pathways get steady. When $\beta _{2}$ expands, chaotic practises have been observed where the values of $\zeta _{\max }$ are non-negative, and insignificant recurring regions can be observed at which the information of $\zeta _{\max }$ are negative. Furthermore, as $\zeta _{\max }$ evolves bigger and closer to 1, the $\zeta _{\max }$ information varies from non-negative to negative, implying that the progressions of the incommensurate DFO-INN system (4.9) transition from inefficient to periodic. We are able to examine the exploding processes of the DFO-INN system (4.9). For $\Im \le -107,$ the DFO-INN framework alongside setting set ($B_{2}$) generates no spiked exertion when $(\beta _{1},\beta _{2})=(1,0.578)$ (see Fig. 6g–i). It demonstrates inconsistent oscillations with explicitly point spikes whenever the energy stimulation is simply increased to $\Im =-105.$ With increasing $\Im$, it changes to a rapid spike in operation, which we revealed via $\Im =-80$ and the additional factors set to their standard setting when $(\beta _{1},\beta _{2})=(0.542,0.578)$ (see Fig. 6j–l).

**Figure 7:**
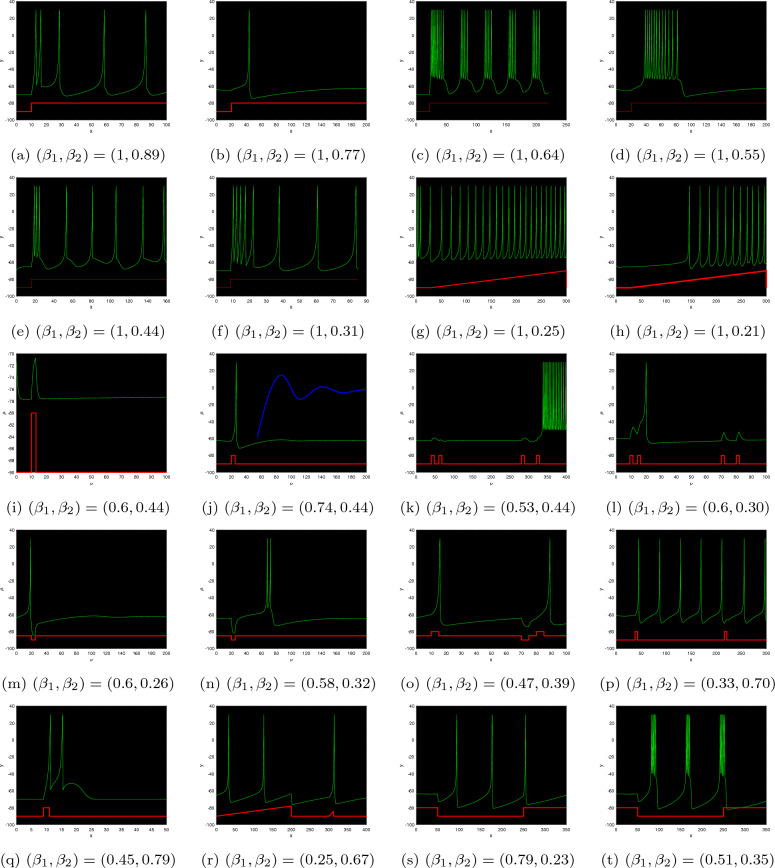
Time evolution and NN activities for incommensurate DFO-INN systems (4.9) for various DFOs and current stimuli $\Im .$.

The data set has become anchored; therefore, we exclusively fluctuate the FO, employing the unchanged energy stimulation. The incommensurate DFO framework exhibits distinctive spiking behaviours for distinct FOs according to the inserted electricity, $\Im$. The prior part discusses the asymptotic robustness of steady-state approaches. In addition to $\Im =-107$, the DFO mechanism’s stable state approach transforms into asymptotically steady for $\beta _{2}<1$. Assume $\Im =-101$ and the asymptotic consistency for a single of the accurate equilibria turns into 0.8120. Fig. 7a–h depicts multiple kinds of resonances when the FOs are $(\beta _{1},\beta _{2})=(1,0.89),~(1,0.77),~(1,0.64),~(1,0.55),~(1,0.44),~(1,0.31),~(1,0.25),~(1,0.21)$. The incommensurate DFO system generates erratic spikes in behaviour. In view of $(\beta _{1},\beta _{2})=(0.83,0.95)$, simply smaller than one, the DFO-INN (4.9) communicates inconsistent sparking. When both FOs reduce to 0.80, it transforms to spiked via barely noticeable fluctuations and yields little spikes (see Fig. 7i–t). In accordance with the aforementioned outcomes, modifications in the incommensurate FOs possess an impact on the fluctuating characteristics of a DFO-INN model with spiking and bursting activities. Additionally, it indicates that an incommensurate DFO could correctly serve the structure’s behaviours, which is reinforced by the phase depictions of the condition components of the incommensurate DFO (4.9) (see Fig. 7).

### Variable DFO

The objective of this subsection is to investigate the intricate behaviour of the DFO-INN in the context of DFVO significance. The framework of the DFVO-INN system is denoted as

4.10 $$\begin{aligned} {\left\{ \begin{array}{ll} \,^{c}\Delta _{\varpi }^{\beta _{1}(\textbf{r})}\textbf{x}(\xi )=0.04\textbf{x}^{2}\big (\textbf{r}-1+\beta _{1}(\textbf{r})\big )-\textbf{y}\big (\textbf{r}-1+\beta _{1}(\textbf{r})\big )+5\textbf{x}\big (\textbf{r}-1+\beta _{1}(\textbf{r})\big )+140+\Im ,~~\textbf{r}\in \mathbb {N}_{\varpi -\beta _{1}(\textbf{r})+1}\\ \,^{c}\Delta _{\varpi }^{\beta _{2}(\textbf{r})}\textbf{y}(\mathfrak {n})=\sigma \big (\eta \textbf{x}\big (\textbf{r}-1+\beta _{2}(\textbf{r})\big )-\textbf{y}\big (\textbf{r}-1+\beta _{2}(\textbf{r})\big )\big ),~~\textbf{r}\in \mathbb {N}_{\varpi -\beta _{2}(\textbf{r})+1}, \end{array}\right. } \end{aligned}$$

where $\beta (\textbf{r})\in (0,1]$ is the DFVO. The DFVO-INN model (4.10) and its numerical system were constructed from Theorem 2.1 in the manner that follows:

4.11 $$\begin{aligned} {\left\{ \begin{array}{ll} \textbf{x}(\textbf{r}+1)=\textbf{x}(0)+\sum \limits _{\mathfrak {n}=0}^{\wp }\frac{\Gamma (\textbf{r}-1-\wp +\beta _{1}(\wp ))}{\Gamma (\beta _{1}(\wp ))\Gamma (\xi -\wp )}\Big (0.04\textbf{x}^{2}(\wp )-\textbf{y}(\wp )+5\textbf{x}(\wp )+140+\Im \Big ),\\ \textbf{y}(\textbf{r}+1)=\textbf{y}(0)+\sum \limits _{\mathfrak {n}=0}^{\wp }\frac{\Gamma (\textbf{r}-1-\wp +\beta _{2}(\wp ))}{\Gamma (\beta _{2}(\wp ))\Gamma (\xi -\wp )}\Big (\sigma \big (\eta \textbf{x}(\wp )-\textbf{y}(\wp )\big )\Big ),~~\textbf{r}=1,2,...~. \end{array}\right. } \end{aligned}$$

In the present moment, we examined the reactions of a system of 1000 independently connected DFVO-INN spikes using various fluctuating trends. For analysing the consequences of DFVO interactions, the system’s procedures for various FOs during a particular value definition are examined. The present study considers an identical type of interconnected system that Izhikevich proposed when he developed the classical integer-order model^8^. The proportion of excitable neurons that inhibit is thought to be 4:1 (80% excitable and 20% hindering neuronal cells). We anticipate employing an analytical framework for developing and simulating a collection of DFVO spikes in NN. Analogous to cortical-in-nature neural networks, it adapts with collaborative interactions and consistent fluctuations. Aside from synapse interactions, every nerve cell in the NN receives unsteady feedback stimulation.

**Figure 8:**
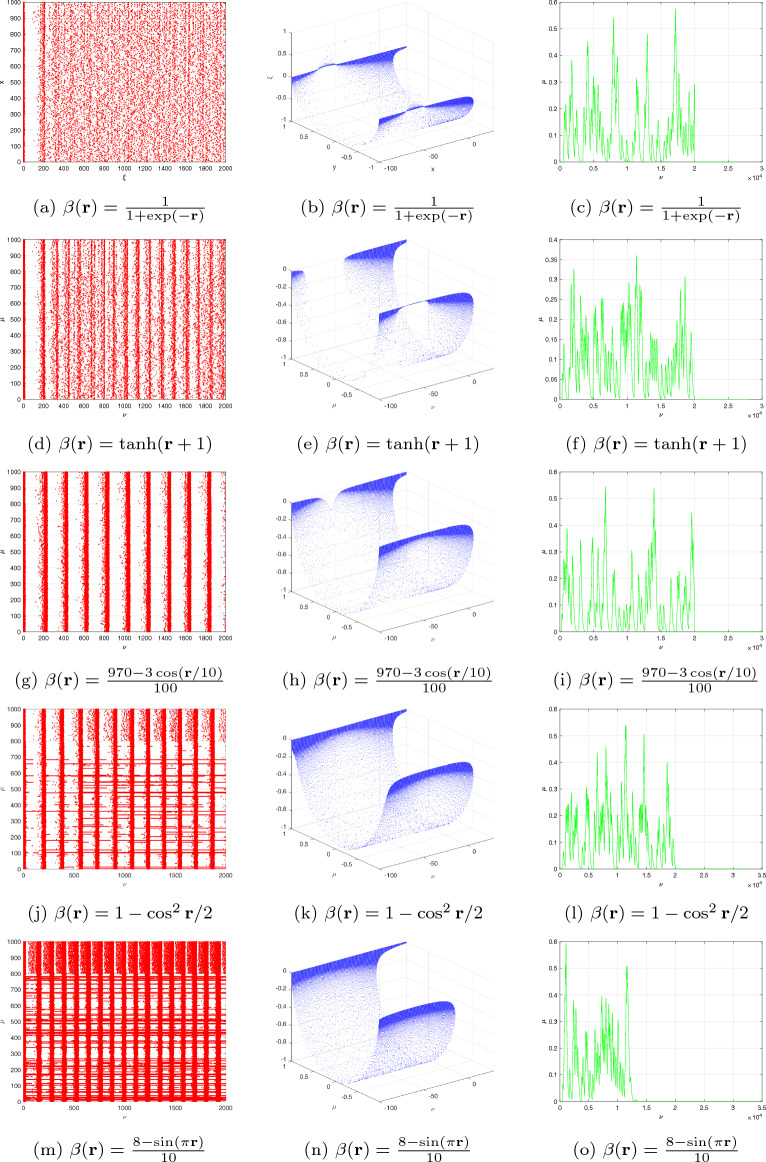
Bifurcation, chaos and $\zeta _{\max }$ behaviour of the neural activities for DFVO-INN system (4.10) generate various kinds of spiking and bursting patterns for various current stimuli $\Im$ using parametric sets ($B_{1}$) and ($B_{2}$) such as (**a**) small spiking; (**b**) synchronized firings; the network started producing cortical-like asynchronous dynamics; (**c**) occasional events of synchronized firings; (**d**) synchronized activity starts disappearing, respectively.

Take into account the DFVO $\beta (\textbf{r})=\frac{1}{1+\exp (-\textbf{r})}$ network with parameter set ($B_{1}$) (see Fig. 8a–c). When $\beta (\textbf{r})=\tanh (\textbf{r}+1),$ then the system exhibits cortical-like asynchronously tempo (see Fig. 8d–f). The intense black robust vertical stripes show that there are actually sporadic synchronized sacking activities (also referred to as alpha regularity ranges)^8^. When the DFVO is changed to $\beta (\textbf{r})=\frac{970-3\cos (\textbf{r}/10)}{100}$, the system’s spiked sequence remains identical in terms of distinctive spike structure (see Fig. 8g–i). Nevertheless, the entire structure is spontaneously interrelated as neuronal cells self-organize into celebrations and develop steady, collaborative interactions. When the DFVO changes to $\beta (\textbf{r})=1-\cos ^{2}\textbf{r}/2$, certain nerve cells in the structure possess greater firing rates than others (see Fig. 8j–l). As the DFVO approaches 1, the processes alter. The succession of terminating behaviours is controlled by approximately fifty percent of the NNs. The synaptic activity vitality within NNs still remains unchanged. As a result, the DFVO patterns modify the spontaneous procedures of the unpredictability ensemble of NNs according to the reaction of the scale-free connection.

Figure 8 illustrates the evolution of the complemented structure when the settings are changed to $\sigma =0.1,~\eta =0.2,~\psi =65$ and $\nu =8$. When $\beta (\textbf{r})=1$, the system exhibits cortical-like instantaneous behaviour. This occurs because the influence of the memory on the cell power and the recuperation factor is fragile for $\beta (\textbf{r})<1.$ The deep black vertical stripes indicate that synchronized explosions occur at certain moments (more commonly referred to as alpha regularity ranges). Gamma patterns are the additional regularity variations. When the DFVFO is $\beta (\textbf{r})=\frac{8-\sin (\pi \textbf{r})}{10}$, the system’s behaviour transforms. The synchronized behaviour vanishes (see Fig. 8m–o). The system’s behaviour diminishes, while certain NNs in the system possess more activity than others. When the fractional order is $\beta (\textbf{r})=1+\exp (-\textbf{r})$, the process entirely shifts The neuronal behaviour structure is controlled by a few neurons in general. The remaining neuronal cells in the cellular structure show no spiked processes. The spiked trend and raster-based sketch closely resemble the scale-free NN. Additionally, when contrasting the findings of the commensurate DF-INN system (4.9) illustrated in Fig. 8 and the outcomes of the incommensurate DF-INN system (4.7) displayed in Fig. 6 the illustrations are unambiguously distinct, indicating that the DFVO influences the dynamical features of the DFVO-INN model (4.9).

## Controlling dynamics of DFO-INN system

### Stabilization of DFO-INN system

Here, the formulation of control procedures that accomplish equilibrium is a crucial component in the research of chaotic frameworks, whether over a discrete or continuous period of time. In the following subsections, we will suggest three separate unpredictable control principles for stabilizing the formerly provided DF-INN system. Whenever we describe stabilization, we indicate incorporating an entirely novel dynamic value $\eta (\xi )$ to equalize all of the technique’s assertions and figuring an efficient responsive equation for such parameters that brings the mechanism stipulated to zero in a reasonable amount of time.

#### Theorem 5.1

Assume that the FO-INN model (3.1) can be controlled using the one-dimensional control principle as follows:

5.1 $$\begin{aligned} \eta _{\textbf{x}}(\xi )=\frac{1}{2}\textbf{x}(\xi )-0.04\textbf{x}^{2}+\textbf{y}-5\textbf{x}-140-\Im . \end{aligned}$$

#### Proof

The time-dependent regulate component $\eta _{\textbf{x}}(\xi )$ is used in the regulated FO-INN, which can be determined by

5.2 $$\begin{aligned} {\left\{ \begin{array}{ll} \,^{c}\Delta _{\varpi }^{\beta }\textbf{x}(\xi )=0.04\textbf{x}^{2}(\xi -1+\beta )-\textbf{y}(\xi -1+\beta )+5\textbf{x}(\xi -1+\beta )+140+\Im -\textbf{x}(\xi -1+\beta +)+\eta _{\textbf{x}}(\xi -1+\beta ),\\ \,^{c}\Delta _{\varpi }^{\beta }\textbf{y}(\mathfrak {n})=\sigma \big (\eta \textbf{x}(\xi -1+\beta )-\textbf{y}(\xi -1+\beta )\big )-\textbf{y}(\xi -1+\beta ). \end{array}\right. } \end{aligned}$$

Plugging the suggested control principle (5.1) into (5.2) produces the straightforward structure

5.3 $$\begin{aligned} {\left\{ \begin{array}{ll} \,^{c}\Delta _{\varpi }^{\beta }\textbf{x}(\xi )=\frac{1}{2}\textbf{x}(\xi -1+\beta )-\textbf{y}(\xi -1+\beta ),\\ \,^{c}\Delta _{\varpi }^{\beta }\textbf{y}(\mathfrak {n})=\sigma \big (\eta \textbf{x}(\xi -1+\beta )-\textbf{y}(\xi -1+\beta )\big )-\textbf{y}(\xi -1+\beta ). \end{array}\right. } \end{aligned}$$

As previously stated, the goal is to demonstrate that the zero equilibrium of (5.3) is asymptotically stable, which indicates that the network’s stipulates coincide with zero over time. The linearization technique, outlined in Theorem 2.2, is capable of helping set up asynchronous reliability. The error mechanism can be produced in the concise form provided by

5.4 $$\begin{aligned} \,^{c}\Delta _{\varpi }^{\beta }\big (x_{1}(\xi ),x_{2}(\xi )\big )=\mho \big (x_{1}(\xi ),x_{2}(\xi )\big )^{\textbf{T}} \end{aligned}$$

produces $$\mho =\begin{pmatrix}0.2&{}-1\\ 0.4&{}-0.2 \end{pmatrix}$$ with $\Im =-104.$ Clearly, it indicates that eigenvalues $\varsigma _{1}$ and $\varsigma _{2}$ of matrix $\mho$ fulfill $\big \vert \arg (\varsigma _{1})\big \vert =\pi >\beta \frac{\pi }{2}$ and $\vert \varsigma _{\iota }\vert <\Big (2\cos \frac{\vert \arg \varsigma _{\iota }-\pi \vert }{2-\beta }\Big )^{\beta }$ for $\iota =1,2$. According to Theorem 2.2, the zero findings of (5.3) is asymptotically stable, and thus the structure is stabilized. $\square$

**Figure 9:**
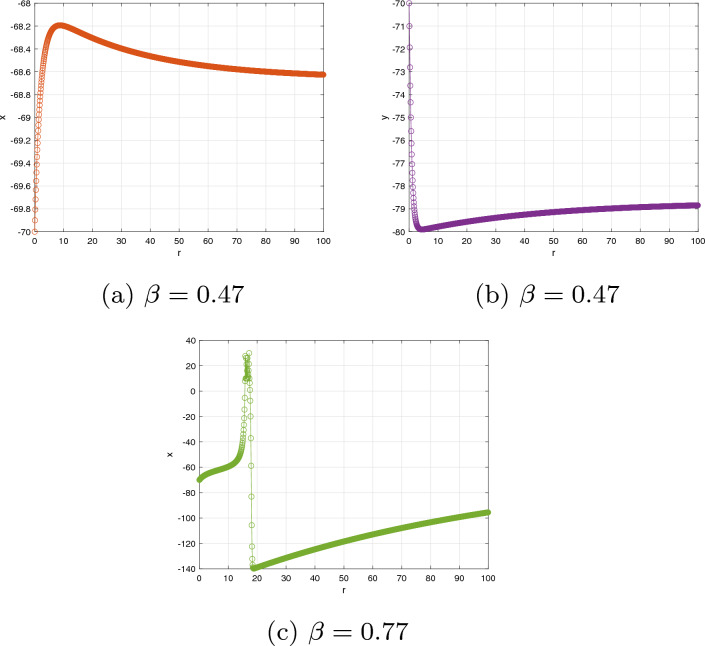
The stabilized depictions of the controlled DFO-INN model (5.3) for set of parameters ($B_{1}$) and current stimuli $\Im =-104.$.

The outcome of Theorem 5.1 are displayed in Fig. 9a–c for $\Im =-104$ and set of parameters ($B_{1}$). Evidently, the regulated mechanism’s declarations merge to zero, as well as the chaotic aspect of the framework, which is removed.

### Synchronization of INN system

An additional intriguing feature, besides the stabilization of DFO- INN, is the synchronization of one chaotic structure with another. The incorporation of an assortment of controlling factors into the regulated chaotic framework and continually modifying the control mechanisms so that the clarifies develop synchronized is referred to as synchronization.

In this section, we will attempt to synchronize a slave DFO framework composed of an amalgamation of the master FO INN system (3.1). The master system will be denoted by the subscript $\mathfrak {m}$ for convenience. The master system is of the following design:

5.5 $$\begin{aligned} {\left\{ \begin{array}{ll} \,^{c}\Delta _{\varpi }^{\beta }\textbf{x}_{\mathfrak {m}}(\xi )=0.04\textbf{x}_{\mathfrak {m}}^{2}(\xi -1+\beta )-\textbf{y}_{\mathfrak {m}}(\xi -1+\beta )+5\textbf{x}_{\mathfrak {m}}(\xi -1+\beta )+140+\Im -\textbf{x}_{\mathfrak {m}}(\xi -1+\beta ),\\ \,^{c}\Delta _{\varpi }^{\beta _{2}}\textbf{y}_{\mathfrak {m}}(\xi )=\sigma \big (\eta \textbf{x}_{\mathfrak {m}}(\xi -1+\beta _{2})-\textbf{y}_{\mathfrak {m}}(\xi -1+\beta _{2})\big )-\textbf{y}_{\mathfrak {m}}(\xi -1+\beta ). \end{array}\right. } \end{aligned}$$

Introducing the slave system as:

5.6 $$\begin{aligned} {\left\{ \begin{array}{ll} \,^{c}\Delta _{\varpi }^{\beta }\textbf{x}_{\textbf{u}}(\xi )=0.04\textbf{x}_{\mathfrak {m}}^{2}(\xi -1+\beta )-\textbf{y}_{\textbf{u}}(\xi -1+\beta )+5\textbf{x}_{\textbf{u}}(\xi -1+\beta )+140+\Im -\textbf{x}_{\textbf{u}}(\xi -1+\beta )\\ \qquad \qquad \qquad +\mathcal {C}_{1}(\xi -1+\beta ),\\ \,^{c}\Delta _{\varpi }^{\beta _{2}}\textbf{y}_{\textbf{u}}(\xi )=\sigma \big (\eta \textbf{x}_{\textbf{u}}(\xi -1+\beta _{2})-\textbf{y}_{\textbf{u}}(\xi -1+\beta _{2})\big )-\textbf{y}_{\textbf{u}}(\xi -1+\beta +\mathcal {C}_{2}(\xi -1+\beta )). \end{array}\right. } \end{aligned}$$

The synchronization regulators are operators $\mathcal {C}_{1}$ and $\mathcal {C}_{2}$. The synchronization oversight for $\textbf{r}\in \mathbb {N}_{\varpi -1+\beta }$ is expressed as

5.7 $$\begin{aligned} \upsilon _{1}(\textbf{r})=\textbf{x}_{\textbf{u}}(\textbf{r})-\textbf{x}_{\mathfrak {m}}(\textbf{r}),\\\upsilon _{2}(\textbf{r})=\textbf{x}_{s_{2}}(\textbf{r})-\textbf{x}_{m_{2}}(\textbf{r}). \end{aligned}$$

The master (5.5) and slave (5.6) systems have been reported to be synchronized if $\lim \limits _{\textbf{r}\mapsto \infty }\vert \upsilon _{\jmath }(\textbf{r})\vert =0,$ for $\jmath =1,2.$ The subsequent results outlines the proposed regulation law for achieving framework synchronization.

#### Theorem 5.2

Consider the system

5.8 $$\begin{aligned} \mathcal {C}_{1}(\textbf{r}-1+\beta ){} & {} =0.04\big (\textbf{x}^{2}_{\textbf{u}}(\textbf{r}-1+\phi )-\textbf{x}^{2}_{\mathfrak {m}}(\textbf{r}-1+\beta )\big )-\big (\textbf{y}_{\textbf{u}}(\textbf{r}-1+\beta )-\textbf{y}_{\mathfrak {m}}(\textbf{r}-1+\beta )\big )\nonumber \\ {}{} & {} \quad +5\big (\textbf{x}_{\textbf{u}}(\textbf{r}-1+\beta )-\textbf{x}_{\mathfrak {m}}(\textbf{r}-1+\beta )\big )-\ell _{1}\upsilon _{1}(\textbf{r}), \end{aligned}$$

where $\ell -{1}\in (-1,2^{\varpi }-1), \jmath =1.$ Then the systems defined in (5.5) and (5.6) are synchronized.

#### Proof

By means of (2.1) and using the error approach stated in (5.7), we have

5.9 $$\begin{aligned} \,^{c}\Delta _{\varpi }^{\beta }\upsilon _{1}(\textbf{r}){} & {} =0.04\big (\textbf{x}^{2}_{\textbf{u}}(\textbf{r}-1+\phi )-\textbf{x}^{2}_{\mathfrak {m}}(\textbf{r}-1+\beta )\big )-\big (\textbf{y}_{\textbf{u}}(\textbf{r}-1+\beta )-\textbf{y}_{\mathfrak {m}}(\textbf{r}-1+\beta )\big )\nonumber \\ {}{} & {} \quad +5\big (\textbf{x}_{\textbf{u}}(\textbf{r}-1+\beta )-\textbf{x}_{\mathfrak {m}}(\textbf{r}-1+\beta )\big )+\mathcal {C}_{1}(\textbf{r}-1+\beta ). \end{aligned}$$

Plugging the control mechanism (5.8) into (5.9), we have

$$\begin{aligned} \,^{c}\Delta _{\varpi }^{\beta }\big ({\upsilon _{1}}(\textbf{r}),{\upsilon _{2}}(\textbf{r})\big )^{\textbf{T}}=\mho \big ({\upsilon _{1}}(\textbf{r}-1+\beta ),{\upsilon _{2}}(\textbf{r}-1+\beta )\big )^{\textbf{T}}, \end{aligned}$$

where

$$\begin{aligned} \mathcal {A}=\begin{pmatrix} -1.2&{}0\\ 0&{}-1.6 \end{pmatrix}. \end{aligned}$$

Since $\varsigma _{1}=1.2~and~\varsigma _{2}=-1.6$ indicate the eigenvalues of $\mho ,$ it is obvious that the eigenvalues $\varsigma _{\iota }.~\iota =1,2$ meets the requirements of Theorem 2.2, the DFO-INN master framework (5.5) and slave model (5.6) are synchronously robust. $\square$

**Figure 10:**
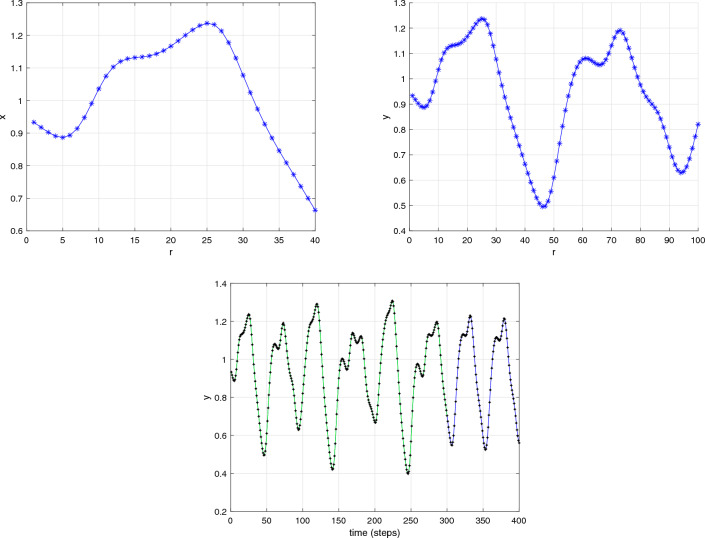
Time-dependent graphs for the fractional error system (5.9) and current stimuli $\Im =4.$.

Mathematical modelling employing MATLAB is used to validate this outcome. We select $\Im =4$ and the ICs stated in^8,49^ with $\epsilon _{1}(0)=-0.01.$ The temporal progression of contends of the fractional oversight mechanism (5.8) dependent on manipulation rules (5.9) is depicted in Fig. 10. It is unambiguous that the deviations are approaching zero, indicating that the synchronization addressed previously is productive.

## Conclusion

In this work, we demonstrated the INN model in the frame of a Caputo-type fractional difference operator. The DFO in the context of commensurate and incommensurate FOs, which serves as the model’s critical parameter, can generate a wealth of bursting and spiking behaviors in the DFO-INN model. The model exhibits inherent bursting oscillations when the fractional order is reduced from 1 (integer-order). As the DFO is reduced further, the oscillations change to irregular spiking or mixed modes. A broad assortment of burst lengths are observed in bursting oscillations when the fractional order diminishes from 1 to increasingly lower values. The model exhibits rapid spiking at significantly smaller FO and VO levels, respectively. The DFOs and injected stimulus current determine the regime of bursting and spiking oscillations. All other parameters remain unchanged, and only the DFO needs to change to shift between regimes. The VO model generates distinct spiking and burst-like oscillations in a sequential manner when other parameters are changed. Additionally, despite any sort of modification input, the model generates spike frequency adaptation that arises from fractional dynamics. The reinforcement mechanism of the memory is responsible for these different oscillations, the spike frequency adaptation, and the entirely experience-dependent spiking behaviors. The stabilization approaches are one-dimensional in nature, which means that we simply need to adapt and control one of the model’s indicators to ensure that all sets tend to zero. The system’s convergence process is predicted employing DFO fixed point theory. Furthermore, we suggest an amalgamated synchronization tactic in which the DFO-INN serves as the master and the slave is an amalgam of the fractional INN. Additionally, the linear modelling approach is used to determine oversight convergence. Analytical findings are included throughout the work to corroborate its results and validate the practicability of the laws suggested. The transition declares of multiple rhythm themes, involving the halting state, are being summarized employing specific fixed parameter distinguishes that correspond to various DFOs $\beta \in (0,1].$

Furthermore, our findings imply that the basic structure of fractional differences provides an overall description of neuronal responses. It is being discovered that FO interactions can be advantageous, if not potent, in the modelling and implementation of contemporary issues^35^. For a futuristic viewpoint, intracellular Gaussian white noise, random interactions for nervous framework currents, and external magnetic induction include regular spiking, chattering, thalamocortical, period-doubling spiking, resonator spiking and chaotic spiking and features of different-designed NNs connected with brain illnesses using DFO interactions will be examined. As a result, more research is essential to explore the attributes of biophysically feasible neuronal cell frameworks and network functioning using Mittag-Leffler kernel behaviour.

## Data Availability

The datasets used and/or analyzed during the current study available from the corresponding author on reasonable request.
